# Supplementary material for: p50-associated COX-2 extragenic RNA (PACER) activates COX-2 gene expression by occluding repressive NF-κB complexes
Source: eLife. 2014 Apr 29;3:e01776. doi: 10.7554/eLife.01776 (PMC4017649; doi:10.7554/eLife.01776)
Supplement: Supplementary file 1. — Sequences of oligonucleotides used in this study DOI: http://dx.doi.org/10.7554/eLife.01776.020 [file elife01776s001.docx]

| **COX-2 primers used in ChIP and RIP experiments** | | |
| --- | --- | --- |
| +9.5 kb | AGAGAAAGACCAGGAGAGTATC | TGGCTTCTCAGTAGCATTCAAC |
| +8.8 kb | AATAAGCTTGTTTGCCATCTTCTG | ATAATCCTTATTCACTGCGACACTG |
| +7.3 kb | GTGATGAGAAGACTGTGTCTCTTAG | CCAATTCAGTAGGTGCATTGG |
| +5.8 kb | TCCAGTAGGCAGGAGAACATATAAC | CCAGATGCCATCTTTGGTGAAAC |
| +4.3 kb | ACACTTGAGTGGCTATCACTTC | GGGTGTTAAATTCAGCAGCAATACG |
| +3.8 kb | CAGCCAGATTGTGGCATAC | GATGATCTACCCTCCTCAAGTC |
| +2.1 kb | AGTCGGGCAATCATCAGG | CCAACTTACAATGCTGACTATGG |
| +0.35 kb | CGGTTAAGTAACTGGCTCATAATG | AAAGAGCTTGGACCGCTAGAG |
| +0.15 kb (‘promoter’) | GGTAGGCTTTGCTGTCTGAG | GTTAGCGACCAATTGTCATACG |
| -0.05 kb | AGAGAACCTTCCTTTTTATAAGACTG | AAAATTTGTGGGGGGTACGAAAAG |
| -0.25 kb | GGGGGTAGTCCCCACTC | AAAAGACATCTGGCGGAAACC |
| -0.45 kb | TGAGCGGCCCTGAGGT | TTCCTCGACCCTCTAAAGACG |
| -0.65 kb | TACATAGCTTTTTAAAAAACTCTTATTTTGTG | GCAACTTAGCTACAAAGATAAATTACAG |
| -0.85 kb | TGTAAATAGTTAATGTGAGCTCCACG | GCAAATTCTGGCCATCGC |
| -1.05 kb | TTTAAAGTTCACTCTTTTGTCTTTTCTG | AAAAGTAAATGCTATGTTGTACTTTGATCC |
| -1.25 kb | CTTTTGTTTAACATCTATCATGGGTAGTG | CACACACACATATACATATATATATTTTTTAGTATC |
| -1.55 kb | CACATCTGATTCTTCATGAGACAC | GAATGATGAAATATGACTAGAGGAGGAG |
| -1.85 kb | GAGAGTACTAAAATTTTACATTACAATCCTG | CAGACAACAGAACAATTAATGTCTG |
| -2.15 kb | AGTGCAAATCTGAGCATAAGG | ATGACCAGCATCCCAAATGTACC |
| -2.45 kb (‘distal’) | AATCTTCCAGAGCTTTCTGTTGAGTTT | TGGCAATCTTCCTAGCATCTTA |
| -2.75 kb | GAAAATGAACAATAAAGACTCTTAATGTCC | GTAGTAGAACCCTGCTCAAAGTAC |
| -3.05 kb | CAAGGTTATGCCATGAAGTTTATCTC | CGTTAGTCCTTTAGTAAAGCCTGATT |
| -3.25 kb | GATCTCCTTTCTTTTTCTCAAATATCTC | CTCATCTGAGTATTATAATGAACAATCAC |
| -3.55 kb | CCTAAAAACAAGTAAAAATCTAAGCAAACTG | ATTTTTTAAGTAGGGTTATGACCTGTC |
|  | | |
| **Primers used in RACE** | | |
|  | initial | nested |
| 5’ RACE | TATGTATGTATGTGCTGCATATAG | ATGTCAGCCTTTCTTAACCTTAC |
| 3’ RACE | GTCTTTGCCCGAGCGCTTCCG | CCGCCGTGTCTGGTCTGTACGTC |
|  | | |
| **Primers used for RNA quantitation** | | |
| COX-2 mRNA | TCTGTACTGCGGGTGGAACA | CAATTTGCCTGGTGAATGATTC |
| PACER | TGTAAATAGTTAATGTGAGCTCCACG | GCAAATTCTGGCCATCGC |
| p50 mRNA | TTGCTGGTCCCACATAGTTG | ATGTATGTGAAGGCCCATCC |
|  | | |
| **Custom siRNA** | | |
| siPACER-753 | TGGAAAGAGAGGCGGGAAA |  |
| siPACER-870 | GGACAAAGGAAGCGGCGAT |  |
|  | | |
| **shRNA** | | |
| PACER ‘870’ shRNA | 5’ - gatcc GGACAAAGGAAGCGGCGAT cttcctgtcaga ATCGCCGCTTCCTTTGTCC tttttg | |
|  | 3’ - g CCTGTTTCCTTCGCCGCTA GAAGGACAGTCT TAGCGGCGAAGGAAACAGG aaaaacttaa | |
